# Biopolymer-coated gold nanoparticles inhibit human insulin amyloid fibrillation

**DOI:** 10.1038/s41598-020-64010-7

**Published:** 2020-05-12

**Authors:** Brahmaiah Meesaragandla, Sanjai Karanth, Una Janke, Mihaela Delcea

**Affiliations:** 1 Institute of Biochemistry, University of Greifswald, Felix-Hausdorff-Straße 4, 17489 Greifswald, Germany; 2 ZIK HIKE - Zentrum für Innovationskompetenz, Humorale Immunreaktionen bei kardiovaskulären Erkrankungen“, Fleischmannstraße 42, 17489 Greifswald, Germany; 3 DZHK (Deutsches Zentrum für Herz-Kreislauf-Forschung), partner site, Greifswald, Germany

**Keywords:** Biophysical chemistry, Proteins, Nanoparticles

## Abstract

Deposits of protein misfolding and/or aggregates are a pathological hallmark of amyloid-related diseases. For instance, insulin amyloid fibril deposits have been observed in patients with insulin-dependent diabetes mellitus after insulin administration. Here, we report on the use of AuNPs functionalized with linear- (i.e. dextrin and chitosan) and branched- (i.e. dextran-40 and dextran-10) biopolymers as potential agents to inhibit insulin fibril formation. Our dynamic light scattering analyses showed a size decrease of the amyloid fibrils in the presence of functionalized AuNPs. Circular dichroism spectroscopy as well as enzyme-linked immunosorbent assay data demonstrated that the secondary structural transition from α-helix to β-sheet (which is characteristic for insulin amyloid fibril formation) was significantly suppressed by all biopolymer-coated AuNPs, and in particular, by those functionalized with linear biopolymers. Both transmission electron microscopy and atomic force microscopy analyses showed that the long thick amyloid fibrils formed by insulin alone become shorter, thinner or cluster when incubated with biopolymer-coated AuNPs. Dextrin- and chitosan-coated AuNPs were found to be the best inhibitors of the fibril formation. Based on these results, we propose a mechanism for the inhibition of insulin amyloid fibrils: biopolymer-coated AuNPsstrongly interact with the insulin monomers and inhibit the oligomer formation as well as elongation of the protofibrils.Moreover, cytotoxicity experiments showed that AuNP-insulin amyloid fibrils are less toxic compared to insulin amyloid fibrils alone. Our results suggest that both dextrin- and chitosan-AuNPs could be used as therapeutic agents for the treatment of amyloid-related disorders.

## Introduction

Deposition of insoluble protein aggregates referred as amyloids has been observed as a common feature of various degenerative diseases (e.g. Alzheimer’s, type II diabetes, Parkinson’s, and Huntington’s disease)^1–5^. Almost 20 different human proteins have been identified to form amyloid deposits and aggregates in intracellular and extracellular matrix of the brain^6^. Amyloid fibrils have been recognized as highly-ordered aggregates, rich in cross β-sheet secondary structures with unbranched filamentous morphology^7^. Generally, amyloid fibril formation involves several steps including oligomers, protofibrils, and fibrils^8,9^. Each of these forms has distinctive molecular conformations and different degrees of toxicity to the neuronal cells^10^.

One of the human proteins where amyloid fibril formation is considered a major problem is insulin. It is a 51-residue polypeptide hormone involved in regulating the blood glucose level and is used for the treatment of diabetes. It consists of an A-chain (21-residues) and B-chain (30-residues) which are connected by a pair of inter-chain disulfide bonds^11^. Furthermore, it has been shown to exhibit an *in vitro* amyloid fibril-forming tendency at certain destabilizing conditions (e.g., low pH, elevated temperature, increased ionic strength, and stirring)^12^. Moreover, insulin amyloid fibril deposits have been observed in patients with insulin-dependent diabetes mellitus after insulin infusion as well as repeated injection at subcutaneous site (injection localized amyloidosis)^13–15^. Insulin amyloid fibrillation is a major concern during insulin manufacture, long-term storage, as well as delivery of the protein and any degree of amyloid fibril formation leads to reduced efficacy of insulin administration^16^.

Currently, there is no approved therapeutic agent available for the treatment of amyloid-related diseases. Recently, there has been an increasing interest in developing nanoparticles (NPs) as therapeutic agents to prevent and treat protein-amyloid related diseases due to their distinctive properties such as: small size, high surface/volume ratio, composition and biocompatibility. It has been shown that NPs may either promote or suppress the amyloid fibrillogenesis. Various copolymer particles such as CeO_2_, TiO_2_, carbon nanotubes, and quantum dots have been reported to promote the rate of amyloid fibril formation *in vitro* depending on the amount and surface of the particles^17^. In contrast, a significant suppression of amyloid fibrillogenesis was observed for hydrophobic teflon and fluorinated NPs^18^.

Gold nanoparticles (AuNPs) have been widely used in biomedical applications as they are chemically inert, readily synthesized, easily functionalized and show excellent biocompatibility^19,20^. However, only very few studies have focused on the influence of AuNPs on amyloid fibril formation of proteins/peptides. Sardar *et al*. have shown that AuNPs inhibit the amyloid fibrillogenesis of β-lactoglobulin in a dose-dependent manner^21^. In another study, Moore *et al*. have studied the effect of AuNPs properties on inhibition of Aβ (beta amyloid) aggregation^22^. They have shown that both surface chemistry and size of NPs can influence the extent of fibril inhibition, whereas the electric charge defines the ability of NPs to alter aggregate morphology. In another study, Guanbin and coworkers have described the distinct size effect of AuNPs and Au nanocrystals (AuNCs) on Aβ fibrillation^23^. In addition, it was found that large AuNPs accelerate Aβ fibrillation, whereas small AuNPs significantly suppress the inhibition process. Esmail *et al*. have used AuNPs to detect the formation of Aβ amyloid fibrils and oligomers^24^. They have demonstrated that the surface plasmon resonance (SPR) band intensity of the AuNPs is sensitive to the presence of oligomers of both Aβ40 and an Aβ40 mutant. In addition, the change in the SPR band intensity can be used to monitor the kinetics of the stable oligomer formation of the Aβ40 mutant.

In this work, we combine spectroscopic and microscopic techniques as well as biological assays to investigate how linear- or branched polymeric ligands at the AuNPs surface influence the insulin amyloid fibril formation. Small-sized AuNPs coated with various biopolymers (dextran-40 (Dex-40), dextran-10 (Dex-10), dextrin (Dxt), and chitosan (Cht)) were used to investigate their effect on insulin amyloid suppression and/or inhibition. The biocompatibility of AuNP-insulin amyloid fibrils was also assessed on human pancreatic and embryonic kidney cell lines.

## Results and discussion

### Synthesis and characterization of biopolymer-coated AuNPs

Biopolymer-coated small-sized AuNPs were prepared using a previously reported protocol^25,26^ with a slight modification. The chosen biopolymers Dex-40, Dex-10, Dxt and Cht as capping ligands present low toxicity, higher dispersibility, availability of functional groups, and robust chemical and thermal stability. Both Dex-40 and Dex-10 molecules are highly branched, whereas Dxt and Cht molecules are linear in nature (see Fig. S1). The branching order is as follows: Dex-40 > Dex-10 > Dxt ≈ Cht.

**Figure 1:**
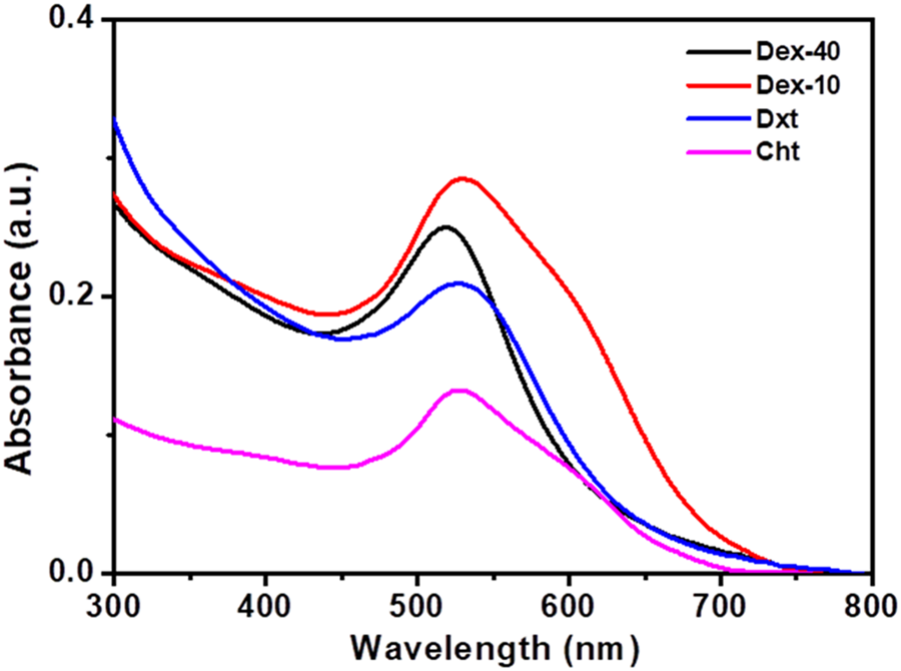
(**A**) UV-Vis absorption spectra of AuNPs coated with Dex-40 (black), Dex-10 (red), Dxt (blue) and Cht (magenta) ligands (AuNPs concentration = 100 nM).

The formation of biopolymer-coated AuNPs was confirmed by the occurrence of SPR bands near 519–528 nm. Figure 1 shows the UV-Vis absorption spectra of AuNPs coated with Dex-40 (black), Dex-10 (red), Dxt (blue) and Cht (magenta) ligands. Highly branched Dex-40-AuNPs showed the SPR band at 519 nm, whereas AuNPs coated with other ligands showed the SPR band at approx. 528 nm. The broadening of SPR band indicates the formation of small particles.

**Figure 2:**
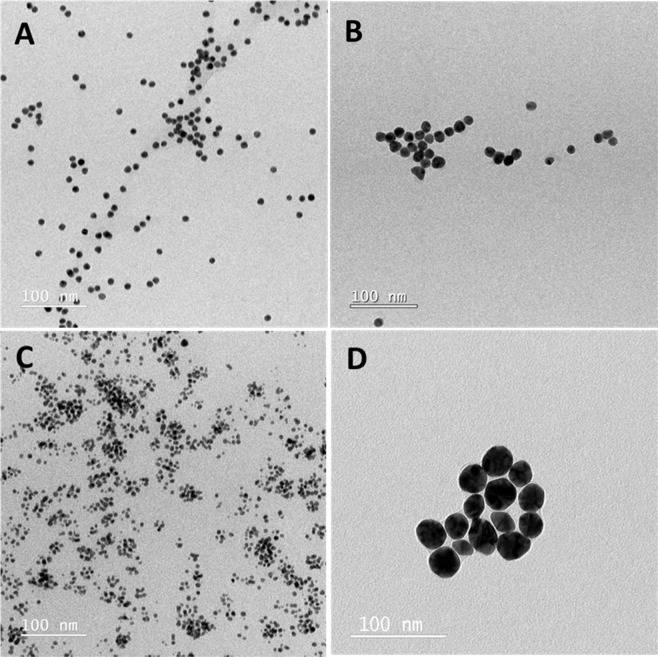
TEM images of AuNPs coated with Dex-40 (**A**), Dex-10 (**B**), Dxt (**C**) and Cht (**D**) ligands.

TEM images shown in Fig. 2 demonstrate that all biopolymer-coated AuNPs have spherical morphology with an average size about 5–15 nm with the exception ofCht-AuNPs which show an average size of about 23.6 ± 5.8 nm.

The hydrodynamic diameters (d_H_) of biopolymer-coated AuNPs measured by DLS was determined to be ∼20, ∼53, ∼40 and ∼55 nm for Dex-40-AuNPs, Dex-10-AuNPs, Dxt-AuNPs and Cht-AuNPs, respectively (Fig. S2A). The overall increase in d_H_ is due to the hydrated expansion of the polymer layer in aqueous state which further varies with length and molecular weight of polymers. Both Dex-10 and Cht-AuNPs show similar d_H_, even though the core size was different as shown in TEM images. This could be due to the multilayer adsorption of polymers on AuNPs surface^27,28^. Among all the biopolymer-coated AuNPs, a large increment in the d_H_ was observed for the Dxt-AuNPs, which is due to the multilayer linear adsorption of Dxt molecules. Moreover, all the biopolymer coatings keep the refractive index of the medium around AuNPs similar as they contain –OH groups at their surface, which may retain the SPR band unaltered^29^. These results indicate that except Dex-40-AuNPs, all the other biopolymer-coated AuNPs exhibits similar SPR band, irrespective of their sizes (see Table S1).”

It has been well distinguished that the maximum wavelength of SPR band for AuNPs strongly depends on size, shape,dielectric environment and space between NPs. Zeta potential data showed positively charged Cht-AuNPs due to the availability of-NH_2_ groups at the surface, whereas all other coated-AuNPs were negatively charged due to the presence of -OH groups at the surface (Fig. S2B).

### Characterization of biopolymer-coated AuNPs at acidic pH and high temperature

In order to understand the effect of both temperature and pH on the stability of biopolymer-coated AuNPs, we have incubated the biopolymer-coated AuNPs in glycine buffer at 65 °C for 3 h. Figure S3 shows the UV-Vis absorption spectra of biopolymer-coated AuNPs in glycine buffer at pH 2 before and after incubation at 65 °C. Both Dex-40 and Dex-10-AuNPs after immediate dispersion in glycine buffer shows a significant change in the SPR band position at longer wavelength in the region of 550–700 nm. However, no detectable change in the SPR band was observed for Dxt-AuNPs, whereas Cht-AuNPs showed an additional peak at 650 nm along with main peak. The appearance of a second peak for Cht-AuNPs is attributed mainly to the bimodal size distribution or aggregation of few particles by decreasing the interspacing between AuNPs.Remarkably, except for Dxt-AuNPs, the SPR band for all biopolymer-coated AuNPs shifted to longer wavelength with decreased intensity after 3 h incubation. Dxt-AuNPs shift the SPR band from 528 to 533 nm without affecting the intensity suggesting aggregation of few particles. The decreased intensity of the SPR band indicates the aggregation of biopolymer-coated AuNPs in acidic medium and at higher temperature. The instability of biopolymer-coated AuNPs in glycine buffer is due to: 1) biopolymer molecules which bind to the AuNPs through weak -OH/-NH_2_ groups; and 2) high ionic strength in the solution which would reduce the electrostatic repulsive forces between AuNPs and further accelerate the aggregation behaviour. In contrast, a small shift (5 nm) in the SPR band for Dxt-AuNPs might be due tothe formation of few aggregates or a slight change in the microenvironment around the NPs as Dxt is thermally stable. The aggregation behavior of the AuNPs was further supported by the increased d_H_ of thebiopolymer-coated AuNPs in DLS measurements (Table 1).As listed in Table 1, the d_H_ of Dex-40-AuNPs, Dex-10-AuNPs, Dxt-AuNPs and Cht-AuNPs after 3 h incubation at 65 °C were determined to be ∼715, ∼566, ∼1866 and 439 nm, respectively. Interestigly, among all the biopolymer-coated AuNPs, Dxt-AuNPs showed a huge increment in d_H_ after 3 h incubation. Contradictorily, a very small shift (∼5 nm)in the SPR band was observed for Dxt-AuNPs even after 3 h incubation (see Fig. S3B). This indicates that the increase in d_H_ for Dxt-AuNPs isdue to either the binding of NPs to the both edges of the linear Dxt molecule or to the formation of the chain-like structures with the NPs. Xing *et al*. showed an increment in the d_H_ of AuNPs after adsorption of siRNA-cathepsin K and a further increase after layering of chitosan and gelatin molecules on the AuNPs surface^30^. According to the zeta potential results shown in Table 1, all biopolymer-coated AuNPs were found to be positively charged at pH 2.0, which could be due to the protonation of -OH groups and availability of -NH^3+^ groups under the acidic condition.

**Table 1:** DLS and Zeta potential results of the biopolymer-coated AuNPs in glycine buffer (pH 2) after 3 h incubation at 65 °C.

AuNPs	d_H_ (nm)	Zeta potential (mV)
Dex-40-AuNPs	715.4 ± 64.6	10.7 ± 2.5
Dex-10-AuNPs	566 ± 75.3	18.6 ± 2.3
Dxt-AuNPs	1866 ± 56.9	1.3 ± 0.3
Cht-AuNPs	439.8 ± 39.6	25.4 ± 0.5

### Interaction of human insulin amyloid fibrils with biopolymer-coated AuNPs

**Figure 3:**
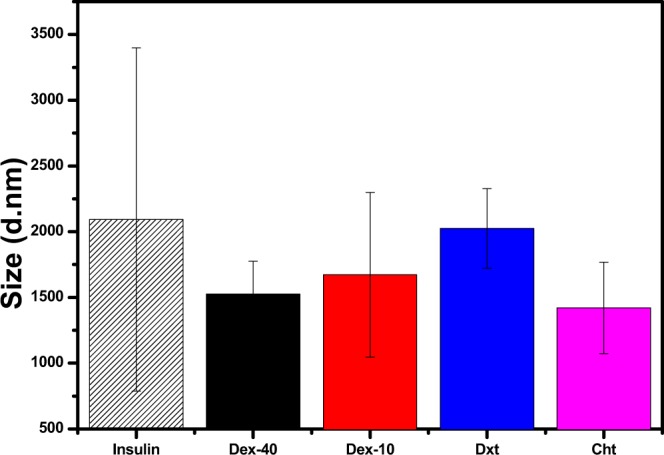
DLS data of pure insulin amyloid fibrils and the same in presence of biopolymer-coated AuNPs in glycine buffer after 3 h incubation at 65 °C (100 nM AuNPs).

Preparation of insulin fibrils at acidic pH and high temperature has been previously described^31^. To study the interaction of biopolymer-coated AuNPs with insulin amyloid fibrils, fibrils were prepared in the presence of biopolymer-coated AuNPs with varying concentrationas described in the experimental section. Initially, DLS analyses were performed to detect the changes in the size distribution of amyloid fibrils in the presence of biopolymer-coated AuNPs. Figure 3 shows the DLS data of insulin solutions incubated in the presence and in the absence of biopolymer-coated AuNPs. Interestingly, insulin alone incubated for 3 h at 65 °C has a d_H_ about 2200 nm, suggesting the formation of long mature insulin amyloid fibrils. The d_H_ of the insulin monomers before heating was about 3.4 nm (Fig. S4) and consistent with previously reported data^32^.

In the presence of biopolymer-coated AuNPs, the d_H_ of insulin amyloid fibrils (ranging from 1500 to 2000 nm) decreased compared to the pure insulin amyloid fibrils. This suggests either formation of oligomers or shortening of mature fibrils caused by the interaction of biopolymer-coated AuNPs with insulin monomers during the fibril formation.

**Figure 4:**
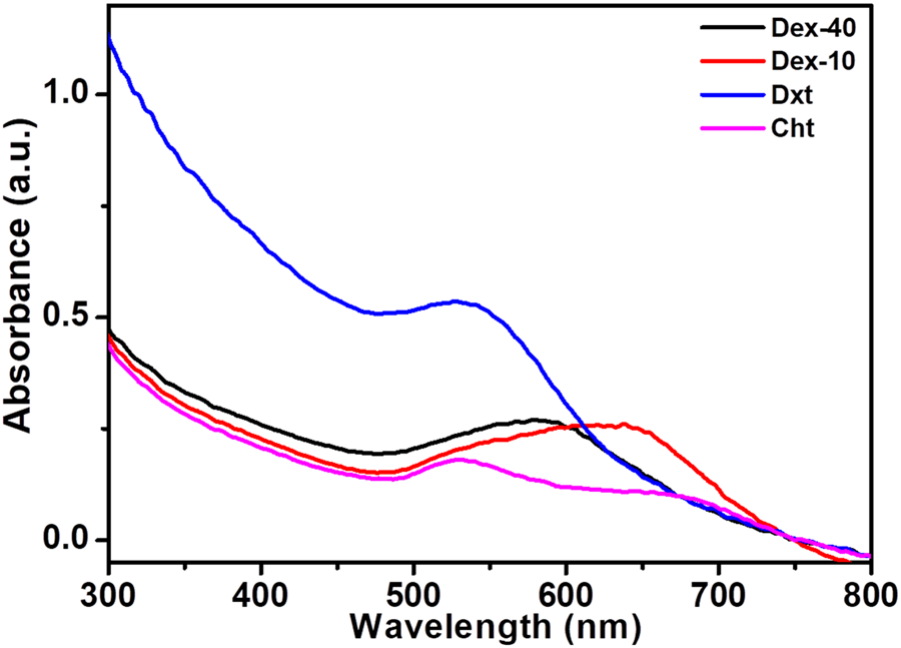
UV-Vis spectra of insulin amyloid fibrils with biopolymer-coated AuNPs in glycine buffer after 3 h incubation at 65 °C (100 nM AuNPs).

To evaluate the effect of biopolymer-coated AuNPson insulin amyloid fibrils, UV-Vis analyses were carried out for biopolymer-coated AuNPs-insulin amyloid fibril solution (final AuNP concentration 100 nM). As shown in Fig. 4, the SPR band position of the biopolymer-coated AuNPs exhibits a change after insulin amyloid fibrils formation. After 3 h incubation, a significant change in the SPR band position was observed for Dex-40 (519 to 590 nm) and Dex-10-AuNPs (528 to 630 nm), whereas no detectable changes were observed for both Dxt- and Cht-AuNPs in the insulin amyloid fibrils.The existence of the SPR band after fibrillation in the presence of insulin indicates the formation of protein corona around the AuNPs, whereas except Dxt-AuNPs, such band (longer wavelength with decreased intensity) was not observed in the absence of insulin (see Fig. S3B). Branched polymers (Dex-40/Dex-10) bind weakly to the AuNPs surface and further, insulin molecules can bind either direct to the AuNPs surface or can intercalate on the AuNPs surface forming protein corona aggregates. Instead, linear molecules (Dxt/Cht) cover the entire AuNPs surface and allow strong binding to the AuNPs. Therefore, insulin can adsorb on the linear polymer coated AuNPs forming a stable AuNP-protein corona.These results indicate that both branched molecules Dex-40 and Dex-10-AuNPs undergo aggregation, whereas the linear molecules Dxt- and Cht-AuNPs are rather stable even after fibril formation. This observation shows that the nature of the biopolymer-coated AuNPs (stable particles or aggregates) has a strong influence on the inhibition of insulin amyloid fibril formation through either strong or weak interaction. The existence of a SPR band for Dxt- and Cht-AuNPs in the presence of amyloid fibrils indicates the stability of AuNPs which can strongly interact with insulin monomers, whereas aggregated AuNPs (Dex-10/Dex-40-AuNPs) can participate in weak interaction with insulin monomers. In the view of stability, both Dxt- and Cht-AuNPs may lead to strong inhibition of insulin amyloid fibrils as they strongly interact with insulin monomers.

We have compared the SPR band position before and after fibril formation to understand the sensitivity of AuNPs during fibril formation. Interestingly, after fibril formation, both Dxt- and Cht-AuNPs resulted in increased intensity of absorption band compared to the Dex-40/Dex-10-AuNPs (see Fig. S5). This suggests that insulin amyloid fibrils make both Dxt- and Cht-AuNPsrelatively stable compared to Dex-40/Dex-10-AuNPs. This could be due to the strong interaction of biopolymer-coated AuNPswith insulin monomers during fibril formation. Moreover, the intact SPR band position before and after fibril formation indicates that the inhibition of fibril formation is purely dependenton the interaction between surface of the biopolymer-coated AuNPsand the insulin monomers. Therefore, AuNPs can be used as a tool to detect the amount of amyloid fibril formation as they exhibit change in the SPR band position and intensity with the fibril formation.

**Figure 5:**
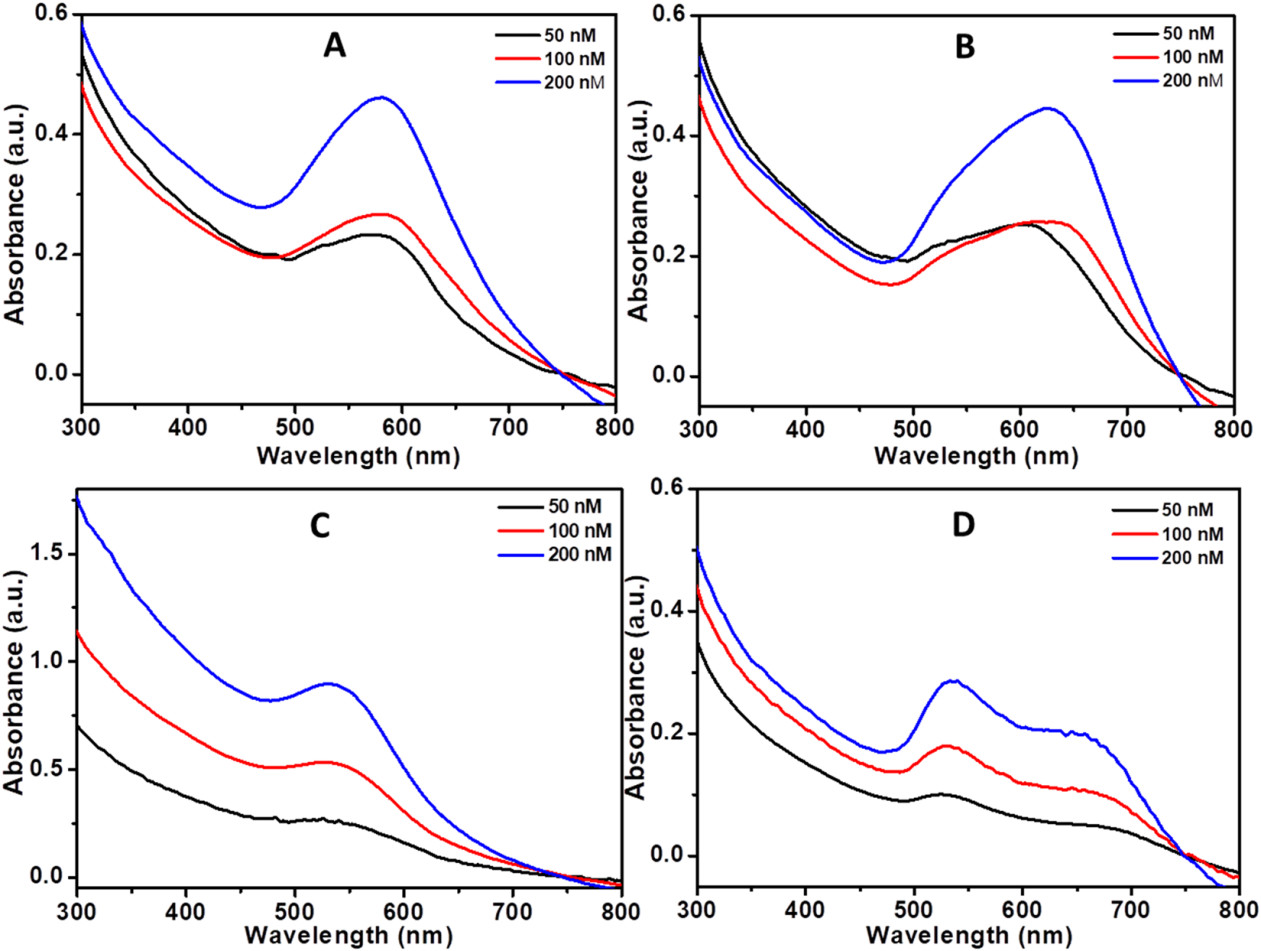
UV-Vis spectra of insulin amyloid fibrils in the presence of biopolymer-coated AuNPs with various concentrations after 3 h incubation at 65 °C in glycine buffer. (**A**) Dex-40, (**B**) Dex-10, (**C**) Dxt, and (**D**) Cht-AuNPs. (AuNPs concentration = 50, 100, 200 nM).

To further understand the effect of biopolymer-coated AuNPconcentration on the insulin amyloid fibril formation, we have performed the absorption measurements for the amyloid fibrils in presence of biopolymer-coated AuNPs at various concentrations. Data showed in Fig. 5 indicates no further shift in the SPR band position upon increasing concentrations. The increase in the intensity of absorption band of biopolymer-coated AuNPs in the insulin amyloid fibrils was purely due to the different concentrations of biopolymer-coated AuNPs.

We have carried out control experiments with bare AuNPs to understand the difference between functionalized and non-functionalized AuNPs interacting with insulin fibrils. We have chosen 10 nm bare AuNPs, which exhibit SPR band at 515 nm (Fig. S6A). Figure S6B shows the UV-Vis spectra of bare AuNPs in the presence of insulin amyloid fibrils before and after 3 h incubation at 65 °C. After immediate dispersion of bare AuNPs to the insulin in glycine buffer, the SPR band shifts towards longer wavelength (542 nm), which is due to the formation of AuNP-insulin protein corona aggregates. This is expected because of the availability of bare AuNPs surface to bind to free -SH, and/or -NH_2_ or -COOH groups of the insulin monomers. This results in the change in the dielectric environment around the AuNPs surface, which allows a shift in the SPR band position. However, a further shift in the absorption band was observed to a longer wavelength (565 nm) after 3 h incubation at 65 °C. This could be due to the aggregation of AuNPs induced by the formed soft corona, which changes their binding sites during fibril formation. There was no further shift in the SPR band position after incubation with biopolymer-coated AuNPs (see Fig. S5). This clearly indicates that inhibition of insulin amyloid fibrils is purely due to the interaction of biopolymers with the insulin monomers. In addition, no further shift in the absorption band was observed for various concentration of bare AuNPs (see Fig. S6C).”

**Figure 6:**
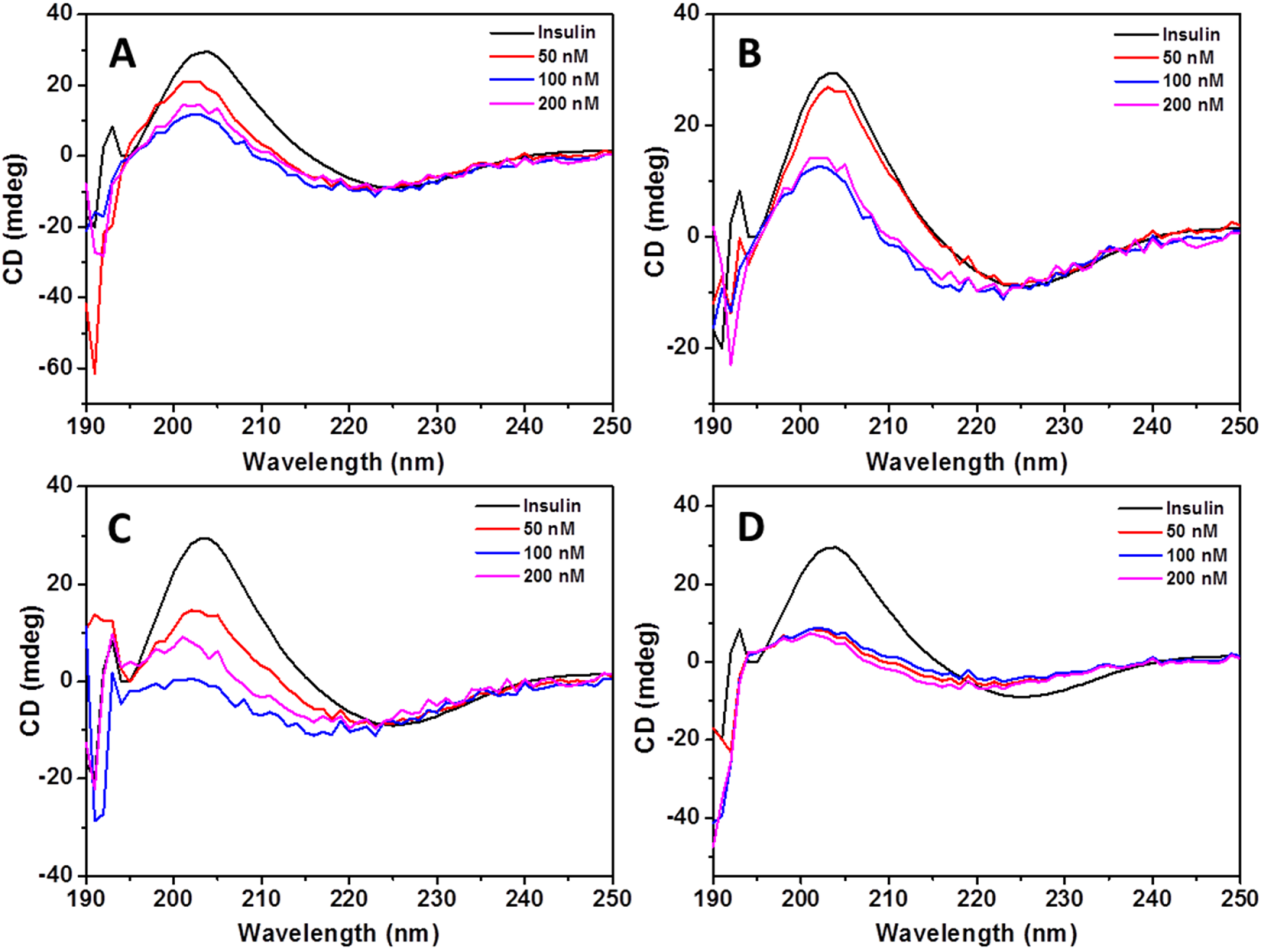
CD spectra of insulin amyloid fibrils in the presence of various concentrations of biopolymer-coated AuNPs after 3 h incubation at 65 °C. (**A**) Dex-40-AuNPs, (**B**) Dex-10-AuNPs, (**C**) Dxt-AuNPs, and (**D**) Cht-AuNPs.

Figures 6 and S7 show the CD spectra of insulin alone and co-incubated with the biopolymer-coated AuNPs with varying concentrations before and after 3 h incubation at 65 °C, respectively. All samples at the beginning of incubation exhibita common spectrum with two negative minima at ∼208 and ∼222 nm, and a pronounced positive peak at ∼195 nm which corresponds to an α-helix-rich secondary structure (Fig. S7). Such peaks were not observed for biopolymer-coated AuNPs alone (see Fig. S8). However, after 3 h incubation, the same samples exhibit typical negative band at 220–225 nm and a positive band at 201–203 nm which corresponds to the mature insulin amyloid fibrils with β-sheet structures (Fig. 6).

The structural changes from α-helix rich to β-sheet structures in CD measurements demonstrated the formation of insulin amyloid fibrils^33^. The intensity and shape of CD spectra were found to be dependent on the type and concentration of AuNPs used during the insulin amyloid fibril formation. The secondary structural transition from α-helix to β-sheet was significantly suppressed by all biopolymer-coated AuNPs. The results indicate that all biopolymer-coated AuNPs diminished the insulin fibril formation in a dose-dependent manner. In particular, both Dxt- and Cht-AuNPs completely inhibit α-to-β transition. Especially, a small amount of Cht-AuNPs inhibit the rich α-helix to β-sheet formation. In contrast, both Dex-40 and Dex-10-AuNPs inhibit moderately β-sheet formation. These results indicate that Dxt- and Cht-AuNPs are more efficient than Dex-40 and Dex-10-AuNPs in inhibiting insulin amyloid fibrillation.

**Figure 7:**
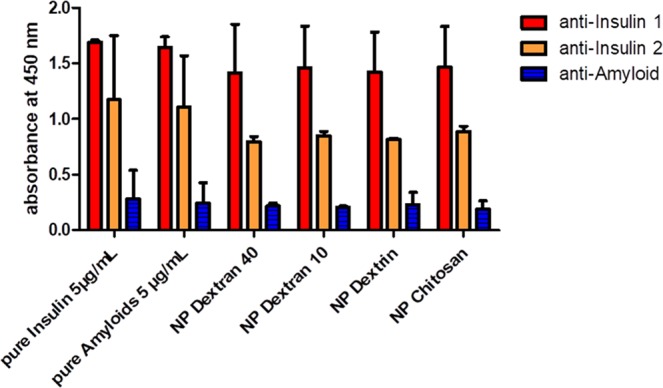
ELISA measurements for insulin, insulin amyloid fibril and AuNP-insulin amyloid fibrils with 10 µg/mL antibodies (anti-Insulin 1, anti-Insulin 2 and anti-amyloid beta antibodies). The bars represent average values of the maxima of the optical density (OD) values. Error bars correspond to the standard deviation.

To further confirm the CD data showing the structural change of insulin after fibril formation in presence of different polymer-coated AuNPs, we have established an ELISA in our laboratory. We have chosen three antibodies (anti-insulin 1, anti-insulin 2 or anti-amyloid beta) to study the interaction with antigens (pure insulin, insulin amyloid fibrils alone and AuNPs-insulin amyloid fibrils). Anti-insulin 1 is the antibody against the insulin receptor binding region, anti-insulin 2 is the antibody against the internal region C-peptide and anti-amyloid beta is the antibody against amyloids in the brain. Figure 7 shows the ELISA measurements of insulin alone before and after fibrillation as well as insulin amyloid fibrils in presence of different polymer-coated AuNPs incubated with anti-insulin 1, anti-insulin 2 or anti-amyloid beta antibodies. It can be seen that anti-insulin 1 antibody binds to all samples (insulin, insulin amyloid fibrils and AuNP-insulin amyloid fibrils) in a similar way. Hardly any change in the binding was observed for all the samples. However, both anti-insulin 2 and anti-amyloid beta antibodies showed difference in the binding with insulin, insulin amyloid fibrils and AuNPs-insulin amyloid fibrils. Anti-insulin 2 antibody binds more strongly to the insulin amyloid fibrils as well as pure insulin, whereas binding has decreased for AuNP-insulin amyloid fibrils. In a similar way anti-amyloid antibody also shows less binding to the biopolymer-coated AuNP-insulin amyloid fibrils than insulin amyloid fibrils alone. Overall, both antibodies showed very less binding to biopolymer-coated AuNP-insulin amyloid fibrils compared to insulin amyloid fibrils alone.

Interestingly, all types of biopolymer-coated AuNPs, regardless of the nature of the attached polymer induced similar binding efficiency of the antibodies. The decrease in the binding efficiency compared to insulin alone might be due to changes in the conformation of insulin from α-helix to β-sheet in the presence of biopolymer-coated AuNPs. However, antibody binding sites might be hidden due to insulin-nanoparticle binding.

From this data it can be proposed that biopolymer-coated AuNPs inhibit the structural changes from α-helix rich to β-sheet structures as indicated by CD data.

**Figure 8:**
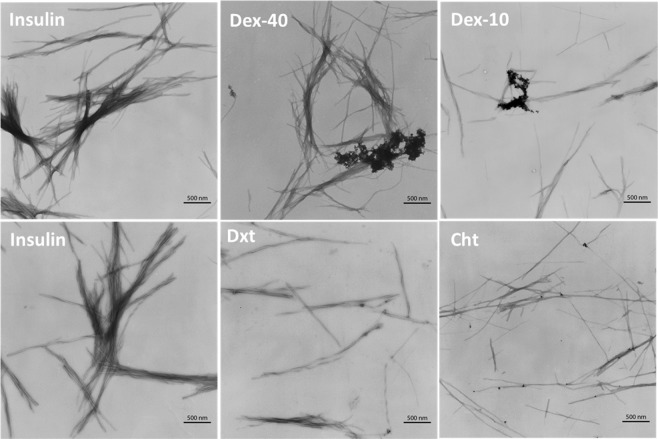
TEM micrographs of insulin sample alone and insulin samples in the presence of Dex-40, Dex-10, Dxt and Cht-AuNPs after 3 h incubation at 65 °C. (AuNPs concentration = 100 nM).

In order to visualize the fibril morphology, TEM analyses were performed on the insulin amyloid fibrils and the same treated with biopolymer-coated AuNPs. Figure 8 shows the TEM micrographs of all insulin samples (in the absence or in the presence of Dex-40, Dex-10, Dxt and Cht-AuNPs) obtained after 3 h of incubation at 65 °C. As expected, high density of thick long fibril aggregates of 10–20 nm in diameter and several μm in length were observed for insulin amyloid fibrils (control), whereas reduced amount of amyloid fibrils with thin and shorter species were observed in the presence of biopolymer-coated AuNPs. However, the level of fibrillogenesis was found to be associated with the type of biopolymer-coated AuNPs. For example, both Dxt and Cht-AuNPs strongly inhibit the amyloid fibril formation compared to Dex-10 and Dex-40-AuNPs. The difference in the fibrillogenesis is due to the interaction of biopolymer-coated AuNPs with the insulin monomers during amyloid fibril formation. In the case of branched polymer-coated AuNPs (Dex-40, Dex-10-AuNPs), the AuNPs undergo self-aggregation which may reduce the interaction with the insulin monomers. This self-aggregationbehaviour was already shown above in the UV-Vis spectra (Fig. 4) and is further confirmed by the aggregation of particles in the TEM images. However, the interaction is different in the case of linear polymer-coated AuNPi.eDxt- and Cht-AuNPs, because both the Dxt- and Cht-AuNPs are rather stable and are attachedon the side of the amyloid fibrils asit can be seen in the TEM images. Therefore, only amorphous insulin amyloid fibril aggregates are formed in the presence of Dex-40 and Dex-10-AuNPs, whereas some short and thin fibrils are observed in the presence of Dxt- and Cht-AuNPs. TEM observations along with CD results clearly demonstrated that AuNPs induce an inhibitory activity towards amyloid fibril formation of insulin.

**Figure 9:**
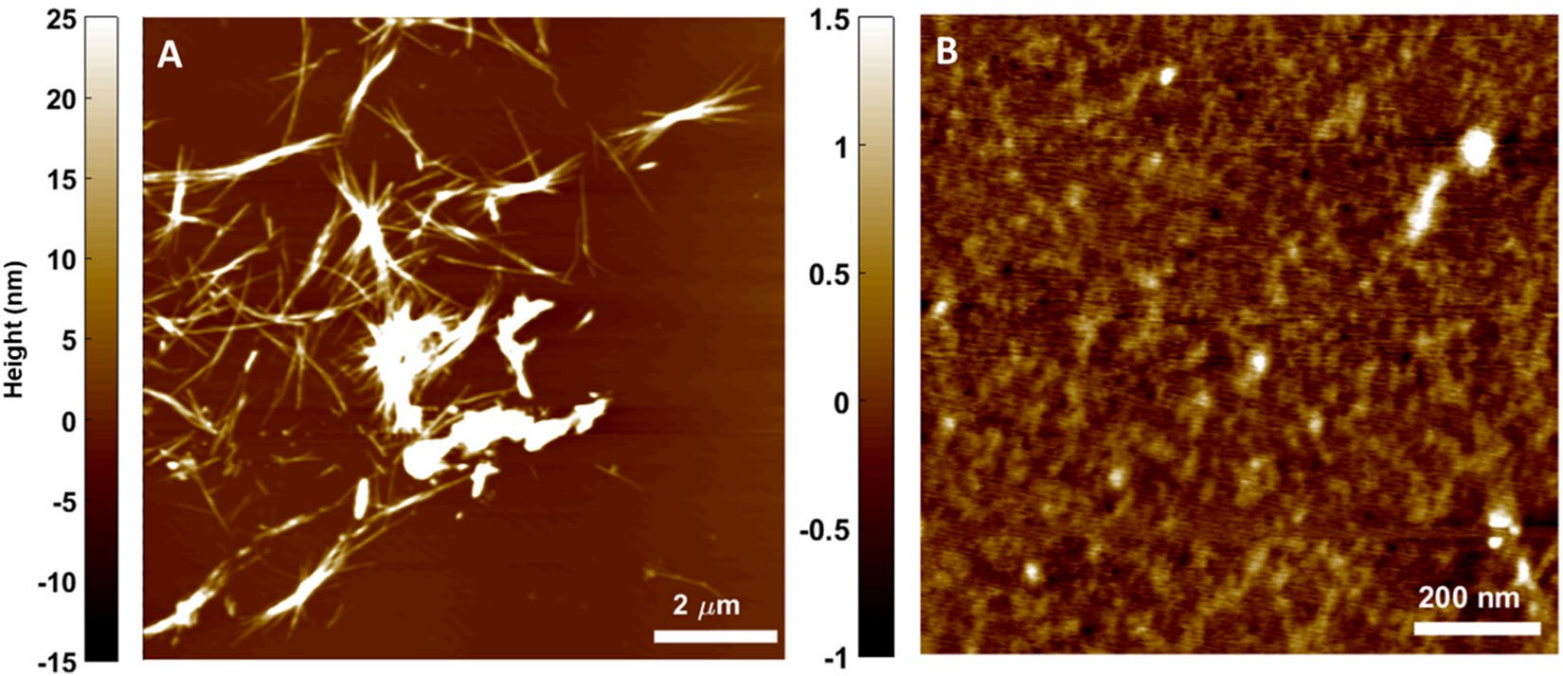
AFM images showing the formation of insulin fibrils (**A**) when subjected to 3 h incubation at 65 °C. Very few oligomers were present (**B**) post fibril formation. The color bar indicates the heights (in nm) of insulin fibrils.

While it was clear from TEM images that fibril thickness was getting reduced, we investigated the faith of disintegrated fibrils and the way their growth was being restricted by biopolymer-coated AuNPs. To understand this, AFM images were taken for the insulin fibrils in the absence and in the presence of biopolymer-coated AuNPs after 3 h incubation at 65 °C. Figure 9 and Fig. S9 show the AFM images of pure insulin before and after 3 h incubation at 65 °C. Insulin monomers before incubation are spherical with height ranging between 0.4 to 0.5 nm (Fig. S9). After 3 h incubation under experimental conditions (pH 2 and 65 °C), the monomers of insulin converted to insulin amyloid fibrils with typical amyloid morphology– long, thick, and unbranched fibrils (Fig. 9A) as well as formation of very few oligomers were observed (Fig. 9B). When observed at smaller scan range, similar height range comparable to that of monomers were visible, but structurally changed to rather strand-like indicating opening of insulin monomers (Fig. 9B).

**Figure 10:**
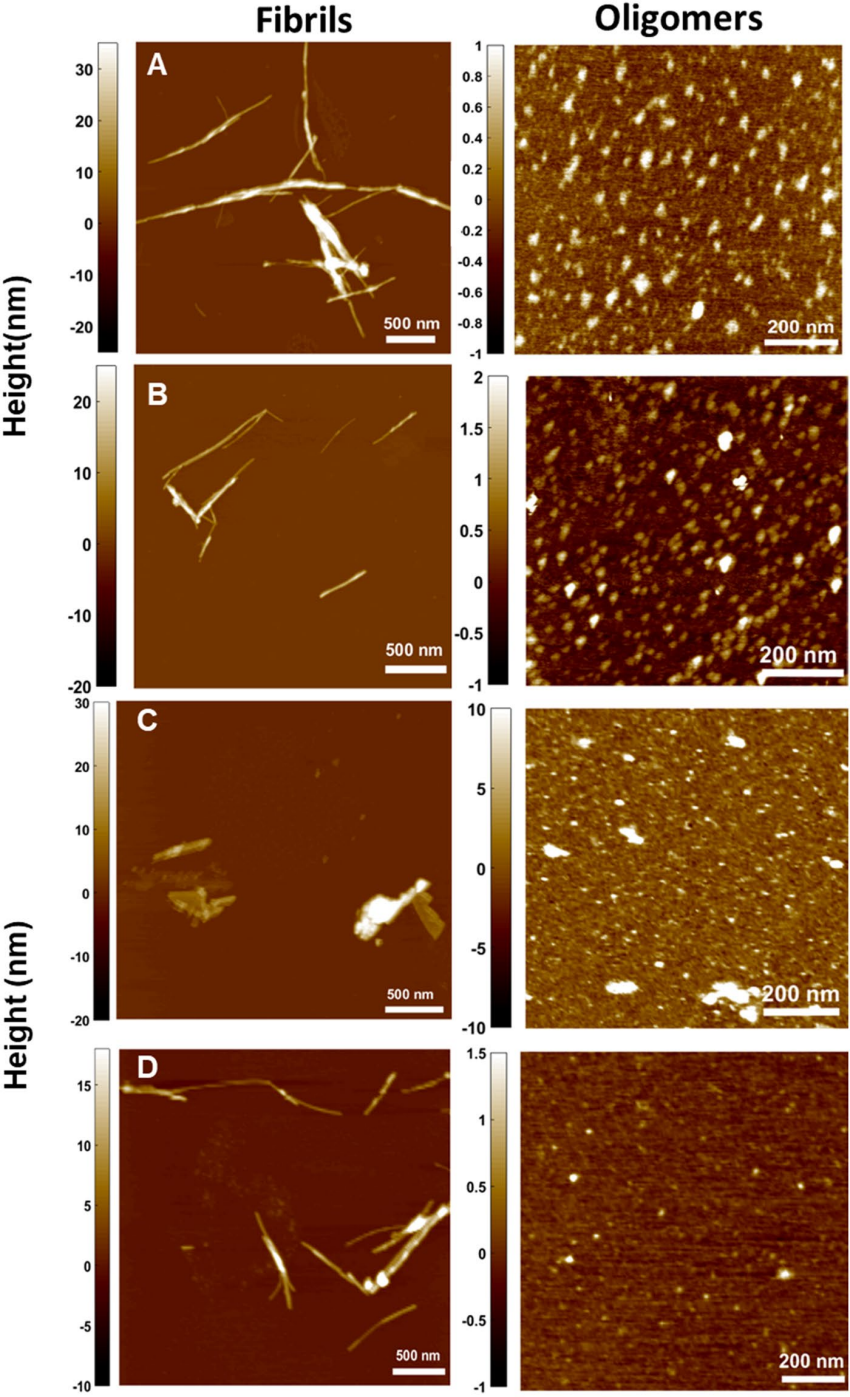
AFM images showing fibrils (left) and oligomers (right) of the insulin samples in the presence of (**A**) Dex-40, (**B**) Dex-10, (**C**) Dxt and (**D**) Cht-AuNPs after 3 h incubation at 65 °C (AuNPs concentration = 100 nM). The color scale on the right represents the height information in nm.

Figure 10 shows AFM images of insulin amyloid fibrils in the presence of biopolymer-coated AuNPs. In the presence of both Dex-40/Dex-10-AuNPs, the insulin fibrils were less prominent and became partially shorter and thinner compared to structures observed for insulin alone after 3 h (Fig. 10A,B). Furthermore, a number of oligomers and globular aggregates with an average height ranging between 0.5–2 nm were observed in the presence of Dex-40/Dex-10-AuNPs (Fig. 10A,B), whereas a very few of such oligomers were formed in the case of insulin amyloid fibrils alone. Interestingly, in the presence of Dxt-AuNPs, very few short and thick fibrils as well as aggregates were found (Fig. 10C).

From the AFM images it can be understood that the effect on fibrils in presence of Dxt-coated AuNPs is less predominant when compared to the same prepared with both Dex-40orDex-10-coated AuNPs. Remarkably, Cht-AuNPs induce thin and short fibrils with a very less amount of oligomers (Fig. 10D). This similar behaviour was also observed in TEM analysis. As depicted in the AFM images, following incubation of insulin with biopolymer-coated AuNPs the height of the fibrils became smaller compared to fibrils formed in the absence of biopolymer-coated AuNPs. The height of insulin fibrils was between 15–20 nm and 10–15 nm in the absence and presence of biopolymer-coated AuNPs, respectively (heights of multiple overlapping fibrils were not considered). The amount of insulin fibril aggregates was significantly predominantly reduced for Dxt/Cht-AuNPs compared to that observed for Dex-40/Dex-10-AuNPs.Overall, biopolymer-coated AuNPs induce shorter fibrillar species, and non-fibrillar aggregates in the insulin sample solutions as compared to the pure insulin fibrils. The obtained results indicate that the surface of biopolymer-coated AuNPs is the main driving factor on insulin amyloid fibrillogenesis.

### Proposed model for the inhibition of insulin amyloid fibrils by biopolymer-coated AuNPs

The amyloid fibrils formation is a multi-step process, involving nucleation, growth and proliferation steps^34^. In general, protein monomers in the nucleation period form oligomers/protofilaments first and further undergo protofibril formation followed by elongation to form mature fibrils. The inhibitory activity of biopolymer-coated AuNPs in the insulin amyloid fibril formation is attributed to the interactions between the surface of the AuNPs and insulin monomers during the nucleation period. The basic structure of all amyloid fibrils involves the polypeptide as main chain and is predominantly stabilized by hydrogen bonds. Moreover, it has been shown that the fibril surface is strongly heterogeneous, and their surface is equally distributed with carboxyl, amino and imino groups^35^. We believe that both functional groups and structural properties of the biopolymers (Dex-40/Dex-10/Dxt/Cht) can inhibit amyloid fibril formation. All dextran family molecules (Dex-40/Dex-10/Dxt) are rich in -OH groups, whereas chitosan molecules have both -OH and -NH_2_ groups. Moreover, Dex-40 and Dex-10 molecules are branched in nature and Dxt and Cht molecules are linear in nature. Having realised that both biopolymer-coated AuNPs and insulin molecules distribute positive charges over their surface at acidic pH, we suggest that electrostatic repulsion and hydrogen bonding are likely to be responsible for the interactions between insulin and biopolymer-coated AuNPs. Generally, proteins fold into a globular structure in which polypeptide chain and the hydrophobic residues are hidden in the core of the protein. When insulin molecules are exposed to certain denaturing conditions (low pH and high temperature), the peptide backbone is not accessible to form the interchain hydrogen bonds which results in the unfolded structure. At that stage, the surface of the biopolymer-coated AuNPs interacts with the insulin monomers and repels their folded structure to completely unfolded structure. Most probably, at the nucleation stage, both Dxt and Cht-AuNPs strongly interact with the insulin monomers *via* their respective -OH and -NH_2_ groups and inhibit the oligomer formation as well as elongation of the protofibrilsand thus,lead to formation of thin and short fibrils. This was supported by the observation of oligomers in the AFM analysis. Moreover, increased intensity of absorption band after fibril formation indicates that both Dxt and Cht-AuNPs particles are quite stable and allow strong interaction with insulin monomers. In another case, Dex-40 and Dex-10-AuNPs undergo self-aggregation which reduces the interaction with insulin monomers and allows the formation of a higher number of oligomers and protofibrils than mature fibrils. This is supported by the aggregation of biopolymer-coated AuNPs in TEM images as well as shift in the absorption band after fibril formation. Although all dextran family molecules have -OH groups, Dxt-AuNPs inhibit insulin amyloid fibrils stronger than Dex-40/Dex10-AuNPs. This could be due to differences in the interaction between biopolymer-coated AuNPs and insulin monomers as they are different in structure (linear and branched). In addition, during the fibrillation process, both Dex-40 and Dex-10-AuNP aggregates interact weakly with insulin monomers as the availability of reactant sites of AuNP aggregates to insulin is lower, whereas the reactant sites for Dxt-AuNPs are higher, leading to inhibition of amyloid fibrillation. When comparing branched-coated AuNPs, Dex-10-AuNPs inhibit insulin amyloid fibrils formation slightly more than Dex-40-AuNPs. This was supported by a higher decrease in the CD signal for Dex-10-AuNPs compared to Dex-40-AuNPs and a slight variation in the fibrils in microscopic imaging analysis. Our results suggest that inhibition of amyloid fibrillation increases as the branching of the polymers decreases.

**Scheme 1:**
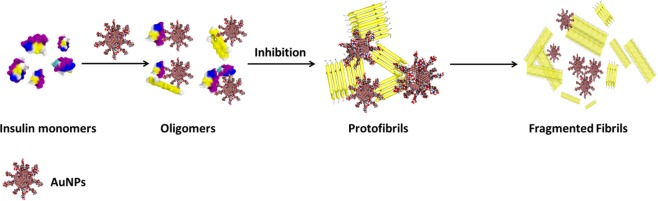
Schematic representation of inhibition of insulin amyloid fibrils in presence of biopolymer-coated AuNPs.

Scheme 1 shows our proposed interaction mechanism of biopolymer-coated AuNPs in the inhibition of insulin amyloid fibrils.

### Cytotoxicity of biopolymer-coatedAuNPs-insulin amyloid fibrils

**Figure 11:**
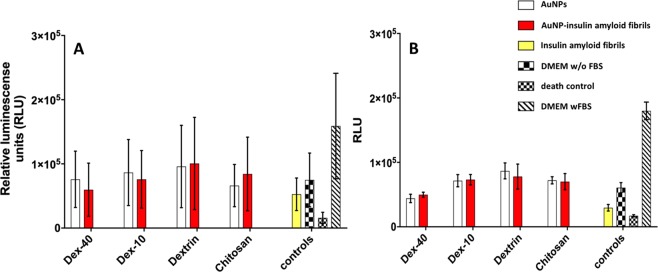
Cytotoxicity results of insulin amyloid fibrils (1 nM, yellow), AuNP (white) and AuNP-insulin amyloid fibrils (red) with PaTu-S (**A**) and PaTu-T (**B**) cells. Error bars correspond to the standard deviation.

Proceeding further, we also investigated the toxicity of insulin amyloid fibrils and biopolymer-coated AuNPs-insulin amyloid fibrils on pancreatic (PaTu-T and PaTu-S) and HEK293 cell lines. Figure 11 shows the cell viability after treatment with different biopolymer-coatedAuNPs, insulin amyloid fibrils and AuNP-insulin amyloid fibrils for PaTu-T and PaTu-S cells. Compared to pure insulin amyloid fibrils, all other types of biopolymer-coated AuNPs and AuNP-insulin amyloid fibrils present a lower cytotoxicity. Stronger luminescence signal indicates higher amounts of vital cells. The increased viability for AuNP-insulin amyloid fibrils compared to the insulin amyloid fibrils is due to the formation of fragmented fibrils or filaments which are less toxic to the pancreatic cells than mature fibrils. Similar behaviour was also observed for HEK293 cells (see Fig. S10). Additionally, to avoid any unspecific interactions of fetal bovine serum (FBS) with biopolymer-coated AuNPs, cells were incubated with media without FBS. However, cells incubated without FBS are growing less than those incubated with FBS, most probably due to missing growth factors and other metabolic requirements.

## Conclusion

We have described the effect of biopolymer-coated AuNPs on the inhibition of insulin amyloid fibrils. Small-sizedAuNPs were prepared using branched and linear biodegradable polymers (Dextran-40, Dextran-10, Dextrin and Chitosan) as stabilizing ligands. We found that the inhibition of insulin fibril formation was dependent on both surface and concentration of biopolymer-coated AuNPs,irrespective of their core sizes. DLS analysis showed a decrease in the diameter of the fibrils in the presence of AuNPs compared to pure insulin amyloid fibrils. UV-Vis analysis indicated the interaction of AuNPs with insulin molecules. Furthermore, CD results demonstrated that the AuNPs strongly inhibit structural changes from α-helix rich to β-sheet structures in a dose-dependent manner, which was further confirmed by ELISA. Shorter and fewer fibrils observed in TEM and AFM images also support the fact that AuNPs inhibit the insulin fibrillation from monomers or oligomers to mature fibrils. Cell cytotoxicity results showed that AuNP-insulin amyloid fibrils are less toxic to pancreatic cells as well as HEK cells compared to treatment with insulin amyloid fibrils alone. Also, a mechanism for the inhibitory activity of AuNPs on the insulin amyloid fibrillation process has been proposed. Overall, linear molecules Dxt- and Cht-AuNPs showed the best inhibitory activity compared to branched Dex-40/Dex-10-AuNPs on insulin amyloid fibrils formation, indicating that the inhibitory activity of AuNPs largely depends on the structure of the ligands as well as functional groups at the surface. Our results suggest that both Dxt- and Cht-AuNPs may be used as therapeutic delivery agents to target the insulin association/aggregation.

## Experimental Sections

### Materials

Human insulin (molecular weight 5700 Da), 10 nm bare gold NPs, tetrachloroauric acid (HAuCl_4_), glycine, NaN_3_ and dextrin were purchased from Sigma-Aldrich (Taufkirchen, Germany). Chitosan, dextran-40, dextran-10, EtOH, HCl, and NaOH were purchased from Roth (Karlsruhe, Germany). The concentration of insulin was measured spectrophotometrically using a molar extinction coefficient of 5734 mol^−1^cm^−1^ at 280 nm. All the chemicals were used as received. The water used was purified through an ultrapure water system, Millipore system and Sartorius Stedim Biotech (Göttingen, Germany).

### Synthesis of AuNPs functionalized with Dex-40, Dext−10 and Dxt ligands

200 µl of 19 mM HAuCl_4_ solution was added to 30 mL of 1 w/v % Dex-40 aqueous solution and the mixture was allowed to stir for 30 min at room temperature (RT). Next, 1.5 mL of 0.05 M NaOH was added to the above solution until colour changed from yellow to colourless. Then, the solution was heated up to 95–99 °C and kept for 30 min at that temperature. Further, the mixture was allowed to cool to RT and it was then centrifuged at 4 °C, 20 × g for 30 min and the pellet was redispersed in 20 mL deionized water. The AuNPs were washed three times with deionized water to remove any excess of free ligands in the solution. Same protocol was used to coat AuNPs with Dex-10 and Dxt. In the case of Dxt-coated AuNPs, Dxt solution was first heated up to 60 °C to dissolve the ligand and then cooled to RT and the above mentioned synthetic protocol was performed to make the Dxt-AuNPs.

### Synthesis of Cht-AuNPs

First, 100 mL of 0.2 w/v % Cht solution was prepared in 1% acetic acid and stirred for 30 min to completely dissolve the Cht molecules. Next, 0.125 M HAuCl_4_ aqueous solution was added to the above boiling solution and kept stirring for 30 min.After 15 min, the colour of the solution changed from colourless to ruby red. This indicated the formation of the Cht-AuNPs.Afterwards, the mixture was allowed to cool to RT and centrifuged at 4 °C, 20 × g for 30 min and redispersed in 20 mL deionized water. The AuNPs were washed three times with deionized water to remove unbound ligands in solution.

### Insulin amyloid fibril formation

Insulin amyloid fibrils were prepared by mixing the solution of human insulin (0.1 mg/mL) with various amounts of biopolymer-coated AuNPs to make different concentrations of AuNPs (50, 100, 200 nM) in the glycine buffer (pH 2.0; 100 mM glycine, 100 mMNaCl, and 1.54 mM NaN_3_). Next, the mixtures of insulin-AuNPs sample solutions were incubated for 3 h at 65 °C with a constant stirring at 300 rpm on a stirring plate (Thermo scientific). In order to reduce the possible formation of fibril nuclei in the solution, all insulin samples were freshly prepared prior to each experiment.

### UV-Vis absorption spectroscopy

UV-Vis spectra of biopolymer-coated AuNPs and insulin amyloid fibrils with AuNPs were measured using NanoDrop 2000c spectrophotometer (Thermo Scientific, Germany) in a 10 mm path length cuvette (Brand UV cuvettes, Germany) at 25 °C. The spectra were recorded between 200 to 850 nm.

### Dynamic light scattering (DLS) and zeta potential measurements

The hydrodynamic diameter (d_H_) and the zeta potential for biopolymer-coated AuNPs and insulin amyloid fibrils with the AuNPs were measured using a Zetasizer Nano-ZS (Malvern Instruments, Herrenberg, Germany). Samples were prepared as described above and filtered through a 0.2 μm (for AuNPs) filter followed by equilibration (typically 5 min) at 25 °C. Measurements were taken at detector angle of 173° using a refractive index of 1.45 and absorption about 0.001 for water. Five independent measurements (12 runs per measurement with a run duration of 5 s) were carried out to estimate d_H_ of the samples, with measurement uncertainty indicated as standard deviation. The zeta potential measurements were carried out at 25 °C with 5 min equilibration between each measurement and a voltage of 50 V using disposal DTS1070 cuvettes using sample dispersions in deionized distilled water (biopolymer-coated AuNPs). The reported zeta potential is an average of five independent measurements of 20 runs (each with duration of 5 s).

### Circular Dichroism (CD) spectroscopy measurements

A Chirascan spectrophotometer (Applied Photophysics, Leatherhead, UK) equipped with a thermostatically controlled cell holder (Quantum Northwest, Liberty Lake, USA) was used for CD measurements which were carried out in the region 190 to 260 nm with a bandwidth of 1.0 nm at 25 °C with a speed scan of 15 nm min^−1^ using a 5 mm path length cuvette (Hellma Analytics, Müllheim, Germany). Each CD spectrum represents an average of 5 scans. Each sample, including the insulin alone and samples with insulin and biopolymer-coated AuNPs, was first diluted five times with deionized water, and then subjected to the CD measurement. The final spectra were obtained by subtracting the corresponding AuNPs without insulin contribution from the original spectra.

### Enzyme-linked immunosorbent assay (ELISA)

Microtiter plates (Capitol Scientific, Austin, USA) were coated with 5 µg/µL of insulin or insulin amyloid fibrils or AuNP-insulin amyloid fibrils (1:10) in a coating buffer (15 mM sodium hydrogen carbonate, pH 9.5 [Merck KGaA, Darmstadt, Germany]) and incubated at 4 °C for 12 h, then washed with washing buffer containing PBS (Merck) with 0.05% Tween 20 (AppliChem, Darmstadt, Germany). After blocking for 2 h with 5% milk powder (Safeway, Calgary, Canada) in PBS at 37 °C, the plate was washed five times with washing buffer and 10 µg/mL of antibodies (mouse anti-Insulin 1 and anti-Insulin 2 [antibodies-online GmbH, Aachen, Germany]) were incubated for 1 h at RT. Plates were washed again five times with washing buffer and 70 ng/mL of the specific peroxidase-coupled antibody (anti-rabbit IgG-HRP and anti-mouse IgG-HRP [Jackson immunoresearch laboratories, West Grove, USA]) was incubated at RT for 45 min. After 5 times washing, the plate was incubated with TMB substrate reagent (3,3′, 5,5′Tetramethylbenzidine; BDBioscience, Heidelberg, Germany) for 5 min at RT. The reaction was stopped by 100 µL 0.5 M H_2_SO_4_ (CarlRoth, Karlsruhe, Germany) and optical density (OD) was measured at 450 nm with a Tecan (Tecan group, Männedorf, Switzerland) infinite 200 absorbance reader. For evaluation, the values of a blank control, without primary antibodies, were subtracted from the samples.

### Transmission electron microscopy (TEM)

TEM analysis of AuNPs or AuNP-insulin amyloid fibrils was performed using a negative staining procedure. Samples were allowed to adsorb for 5 min onto a glow-discharged Pioloform carbon-coated 400-mesh grid, which was then transferred onto two droplets of deionized water, and finally for 30 s onto a drop of 1% aqueous uranyl acetate. After blotting with filter paper and air-drying, samples were examined using a transmission electron microscope LEO 906 (Carl Zeiss Microscopy GmbH) at an acceleration voltage of 80 kV. Adobe Photoshop CS6 was used to edit the micrographs.

### Atomic force microscopy (AFM)

AuNP-insulin amyloid fibrils (20 µl, 2.5 μM) samples were spread on atomically flat muscovite mica sheet (Science Services, Germany,used as substrate) having RMS of ~0.1 nm and was allowed to rest for 60 seconds. Then, the substrate was washed for 15 seconds with deionized water and dried in a laminar flow box (ScanLaf Class 2, LaboGene, Lynge, Denmark). Air imaging was performed using NanoscopeIIIa controller (Veeco/Digital Instruments, Santa Barbara, USA). Images were captured in tapping mode using OMCL-AC160TS (Olympus Corporation, Japan) cantilevers having an approximate curvature radius of 10 nm and a spring constant of 42 N m^−1^. Data processing of the obtained AFM images were carried out using a home-written MATLAB (The MathWorks, 2010b, Natick, USA) script.

### Cytotoxicity assay

The determination of cell viability was performed by the CellTiter-Glo 2.0 assay from Promega (Madison, USA) following manufacturer’s instructions. Briefly, 5 × 10^4^ cells/mL were seeded in DMEM media without fetal bovine serum (FBS), to exclude albumin binding to the biopolymer-coated AuNP, and sedimented for 2 h in an opaque walled 96-well plate. After aspiration of media, 1 nM of biopolymer-coated AuNPssolution was added and incubated for 24 h at 37 °C and 5% CO_2_. Further, 100 µl of CellTiter-Glo 2.0 was added to the wells and 10 min after incubation, the luminescence signal was measured in a SpectraMax Paradigm (Molecular Devices, San Jose, USA).

## Supplementary Information

Supplementary Information.
