# Supplementary material for: Biopolymer-coated gold nanoparticles inhibit human insulin amyloid fibrillation
Source: Sci Rep. 2020 May 12;10:7862. doi: 10.1038/s41598-020-64010-7 (PMC7217893; doi:10.1038/s41598-020-64010-7)
Supplement: Supplementary file 1 — Supplementary Information. [file 41598_2020_64010_MOESM1_ESM.docx]

**Supplementary Information**

**Biopolymer-coated gold nanoparticles inhibit human insulin amyloid fibrillation**

Brahmaiah Meesaragandla,^1,2^ Sanjai Karanth,^1,2^ Una Janke,^1,2^ and Mihaela Delcea^1,2,3^*

*^1^Institute of Biochemistry, University of Greifswald, Felix-Hausdorff-Straße 4, 17489 Greifswald, Germany*

*^2^ZIK HIKE - Zentrum für Innovationskompetenz „Humorale Immunreaktionen bei kardiovaskulären Erkrankungen“, Fleischmannstraße 42, 17489 Greifswald, Germany*

*^3^DZHK (Deutsches Zentrum für Herz-Kreislauf-Forschung), partner site Greifswald, Germany*

*Corresponding author, [delceam@uni-greifswald.de](mailto:delceam@uni-greifswald.de)

**Figure S1**. Chemical structures of Dex-40, Dex-10, Dxt and Cht molecules.

**Figure S2**. DLS and zeta potential data of AuNPs coated with Dex-40, Dex-10, Dxt and Cht ligands.

**Table S1**. Surface plasmon resonance (SPR) peak position and size from TEM analysis for AuNPs coated with different ligands.

**Figure S3**. UV-Vis spectra of different biopolymer-coated AuNPs in glycine buffer before and after 3 h incubation at 65 °C.

**Figure S4**. DLS data of pure insulin in glycine buffer before incubation at 65 °C.

**Figure S5**. UV-Vis spectra of insulin amyloid fibrils in presence of Dex-40, Dex-10, Dxt, and Cht-AuNPs in glycine buffer before and after incubation at 65 °C for 3h.

**Figure S6**. UV-Vis spectra of bare AuNPs and the same in the presence of insulin amyloid fibrils before and after incubation at 65 °C for 3h at different AuNPs concentrations.

**Figure S7**. CD spectra of pure insulin and same in the presence of different polymer-coated AuNPs with various concentrations before incubation.

**Figure S8**. CD spectra of different polymer-coated AuNPs.

**Figure S9**. AFM image of pure insulin monomers before incubation.

**Figure S10**. Cytotoxicity effect of different coated AuNPs, insulin amyloid fibrils, and AuNP-insulin amyloid fibrils on HEK293 cells.

**
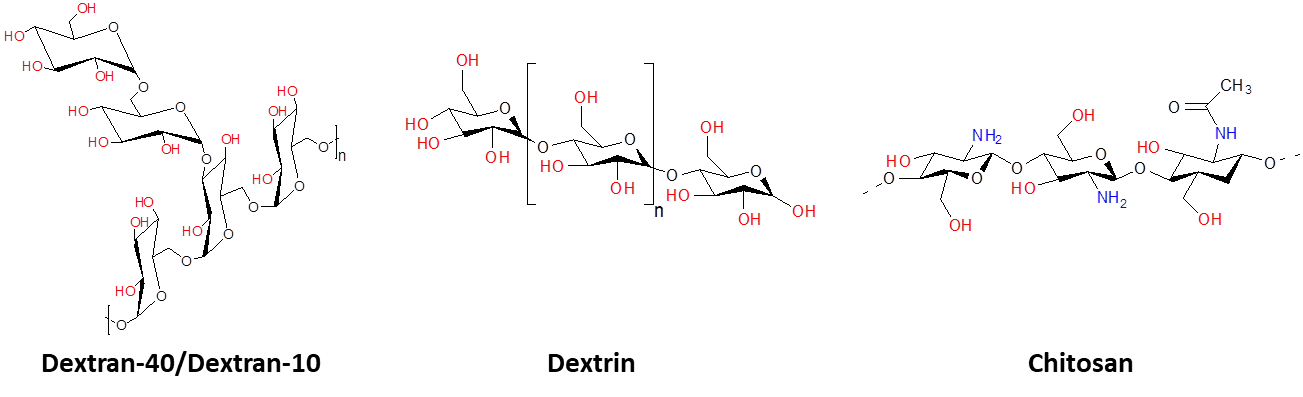
**

**Figure S1.** Chemical structures of Dex-40, Dex-10, Dxt and Cht molecules.


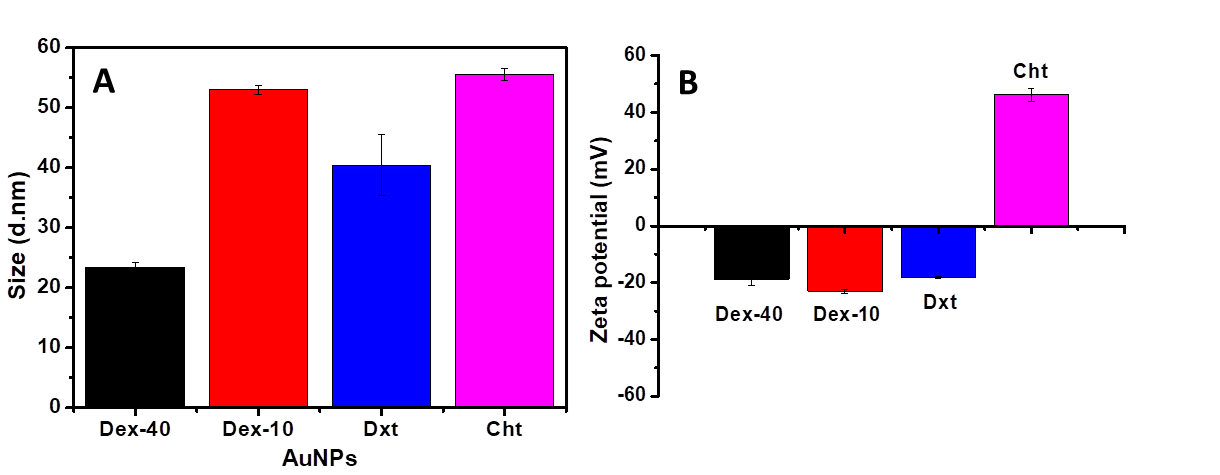


**Figure S2.** DLS (A) and zeta potential (B) data of AuNPs coated with Dex-40 (black), Dex-10 (red), Dxt (blue) and Cht (magenta) ligands.

**Table S1.** Surface plasmon resonance (SPR) peak position and size from TEM analysis for AuNPs coated with different ligands.

| AuNPs | SPR (nm) | TEM (nm) |
| --- | --- | --- |
| Dex-40 | 519 | 8.7 ± 0.6 |
| Dex-10 | 528 | 13.5 ± 2.0 |
| Dxt | 527 | 6.3 ± 2.1 |
| Cht | 528 | 23.6 ± 5.8 |

**
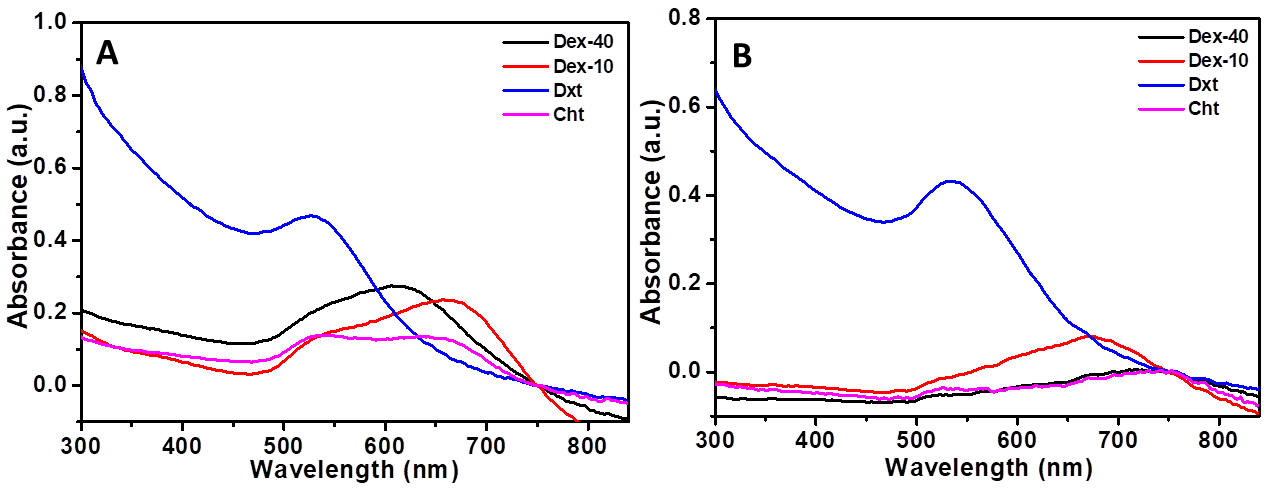
**

**Figure S3.** UV-Vis spectra of different biopolymer-coated AuNPs in glycine buffer A) before and B) after 3 h incubation at 65 °C (100 nM AuNPs).

**Figure S4.** DLS data showing the size of pure insulin in glycine buffer before incubation at 65 °C.


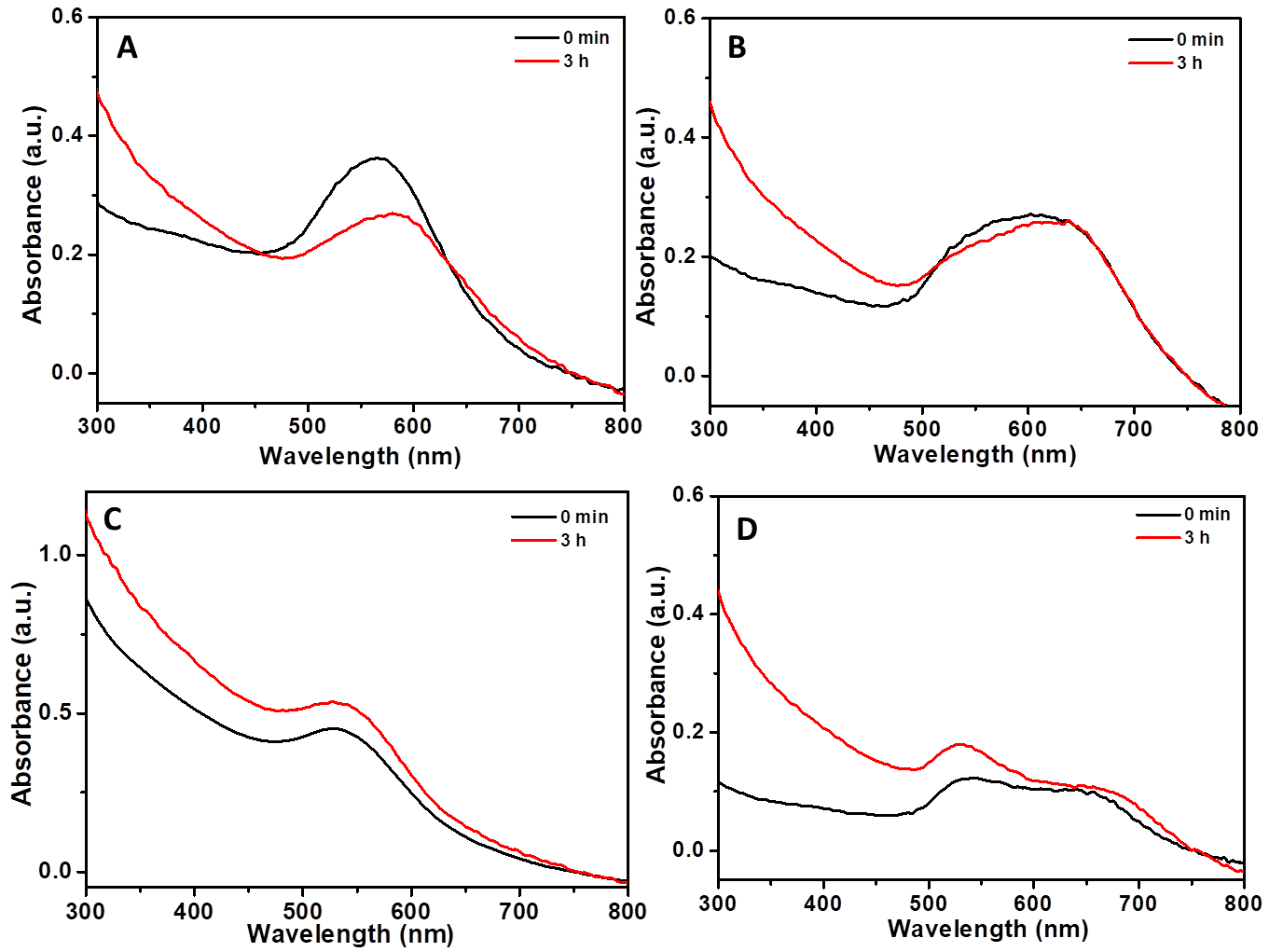


**Figure S5.** UV-Vis spectra of insulin amyloid fibrils in presence of A) Dex-40, B) Dex-10, C) Dxt, and D) Cht-AuNPs in glycine buffer before and after incubation at 65 °C for 3h (100 nM AuNPs).


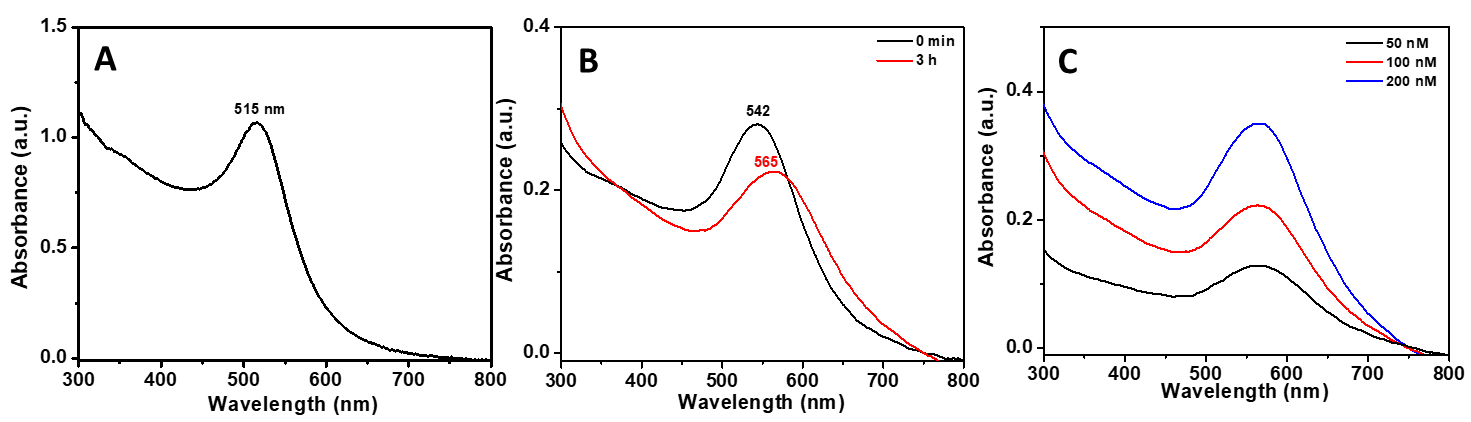


**Figure S6:** UV-Vis spectra of (A) bare AuNPs, (B) AuNPs (100 nM) in the presence of insulin amyloid fibrils, (C) AuNPs (50, 100, 200 nM) in the presence of insulin amyloid fibrils after 3 h incubation at 65 °C.


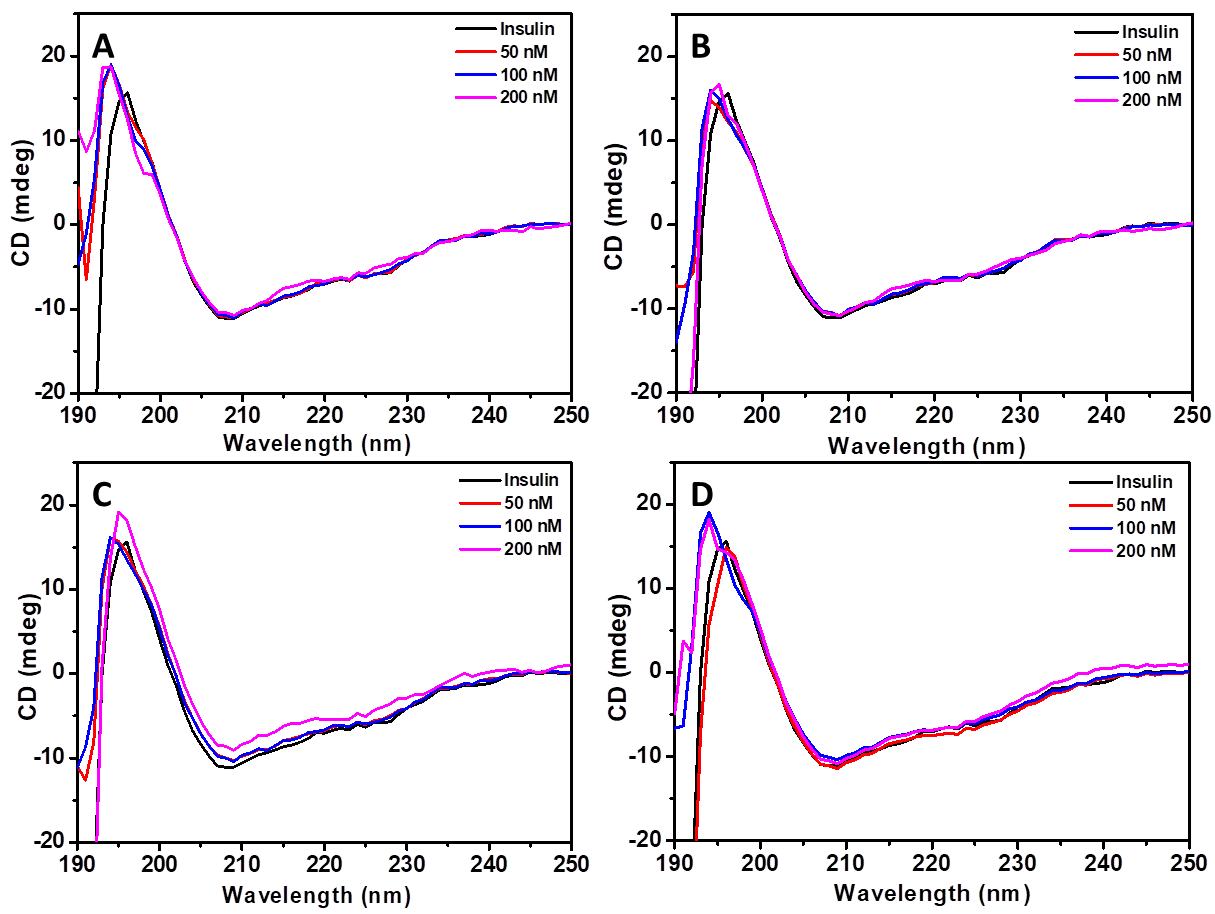


**Figure S7:** CD spectra of pure insulin and same in the presence of different polymer-coated AuNPs with various concentrations before incubation. A) Dex-40-AuNPs, B) Dex-10-AuNPs, C) Dxt-AuNPs and D) Cht-AuNPs.

**
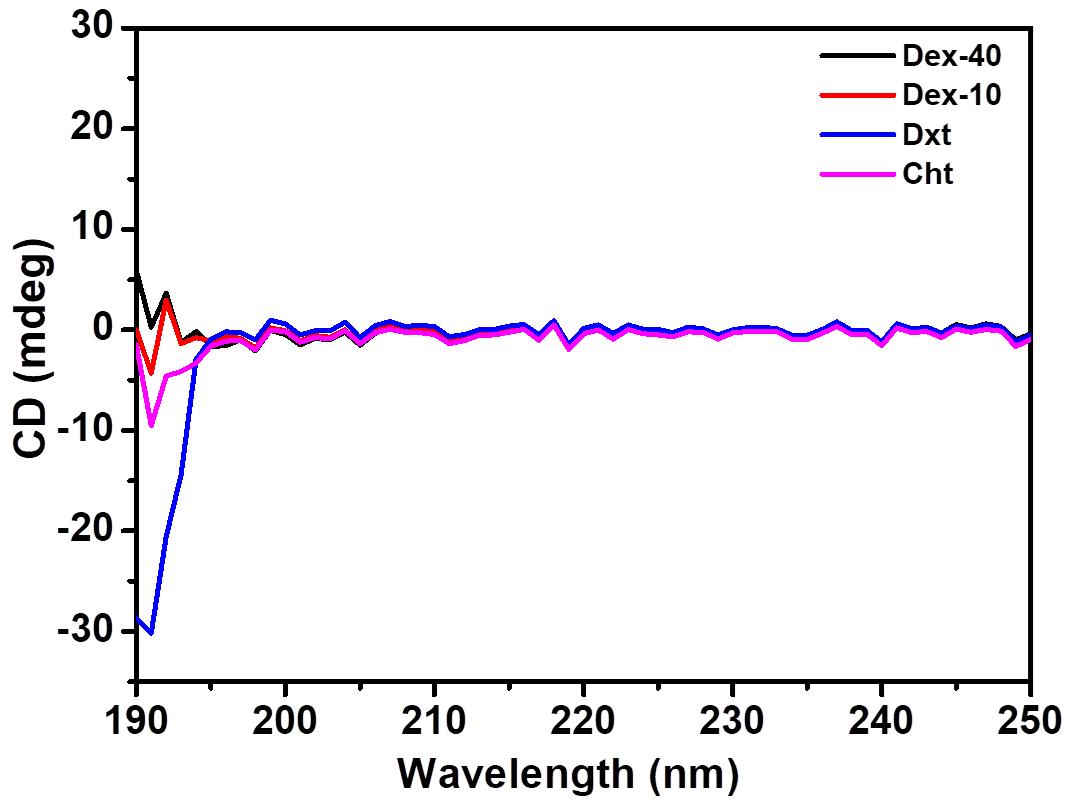
**

**Figure S8:** CD spectra of different polymer-coated AuNPs.

**
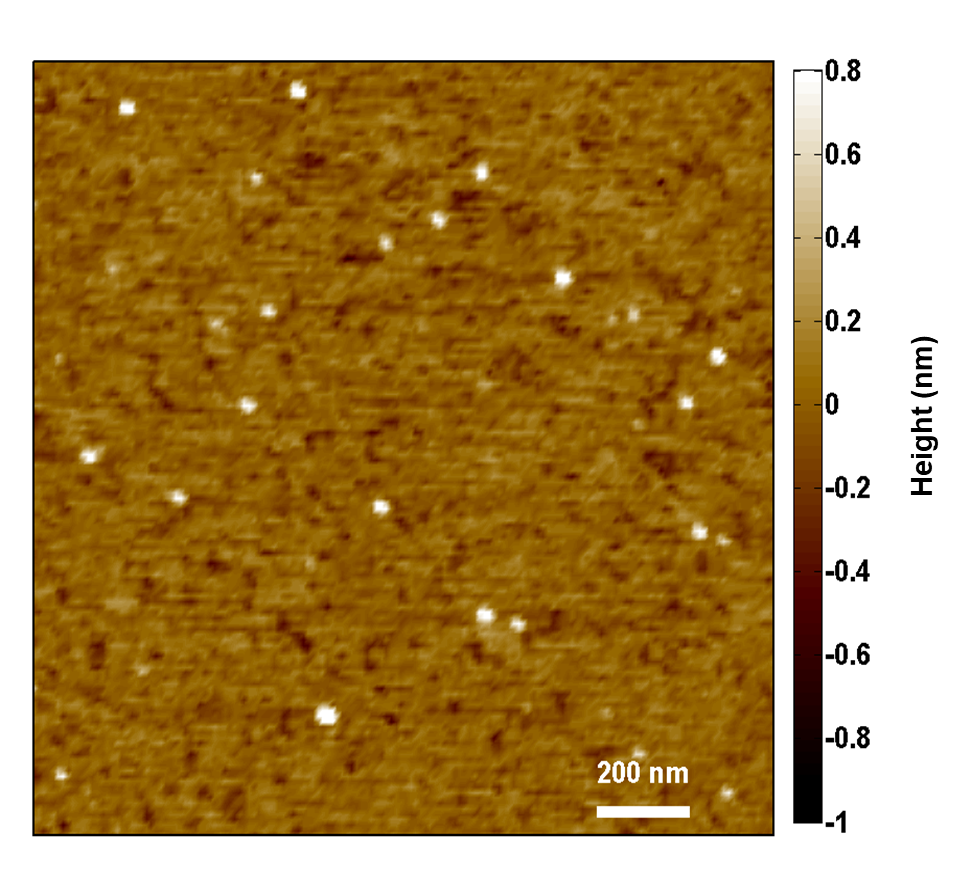
**

**Figure S9**. AFM image of pure insulin monomers before incubation.

**
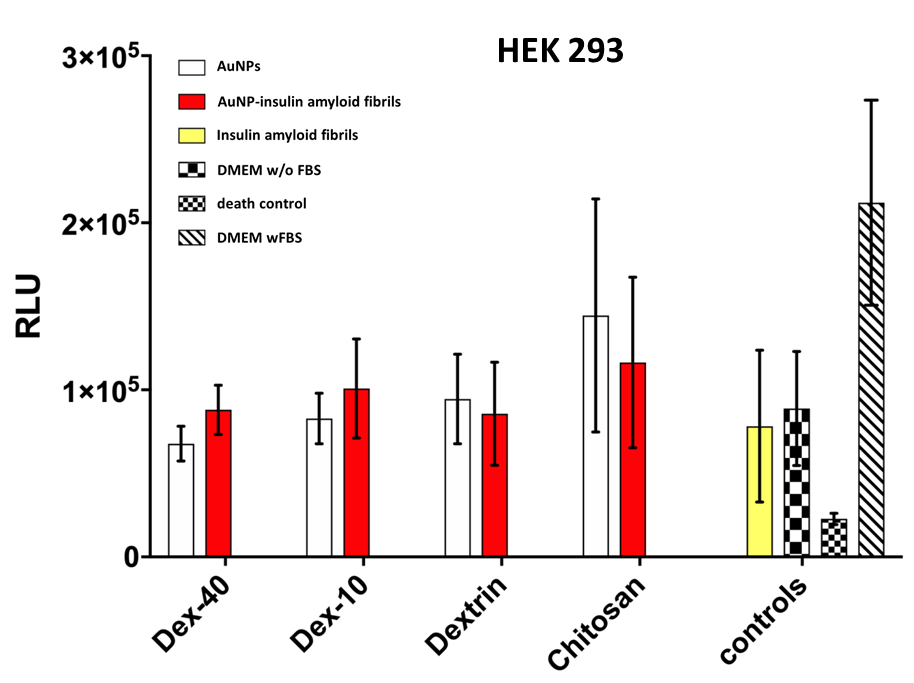
**

**Figure S10**: Cytotoxicity effect of different coated AuNPs, insulin amyloid fibrils, and AuNP-insulin amyloid fibrils on HEK293 cells. Error bars correspond to the standard deviation.
